# Association of the* MDM2* SNP285 Polymorphism with Cancer Susceptibility: A Meta-Analysis

**DOI:** 10.1155/2016/4585484

**Published:** 2016-11-07

**Authors:** Ping Wang, Meilin Wang, Sanqiang Li, Lingjun Ma, Shoumin Xi, Jing He

**Affiliations:** ^1^The Key Laboratory of Pharmacology and Medical Molecular Biology, Medical College, Henan University of Science and Technology, Luoyang 471023, China; ^2^The Molecular Medicine Key Laboratory of Liver Injury and Repair, Medical College, Henan University of Science and Technology, Luoyang 471023, China; ^3^Department of Pediatric Surgery, Guangzhou Institute of Pediatrics, Guangzhou Women and Children's Medical Center, Guangzhou Medical University, Guangzhou 510623, China; ^4^State Key Laboratory of Oncology in South China, Collaborative Innovation Center for Cancer Medicine, Department of Experimental Research, Sun Yat-sen University Cancer Center, Guangzhou 510060, China

## Abstract

The* mouse double minute 2* (*MDM2*) gene encodes a negative regulator for p53, and the polymorphism SNP285 in the promoter region of* MDM2* gene has been implicated in cancer risk, but individual published studies had inconclusive results. Therefore, we performed this meta-analysis to obtain a more precise estimation between* MDM2* SNP285 polymorphism and risk of cancer. A systematic literature search was performed using the PubMed, Embase, and Chinese Biomedical (CBM) databases. Ultimately, 16 published studies comprising 14,573 cases and 9,115 controls were included. Pooled odds ratios (ORs) and 95% confidence intervals (CIs) were calculated to assess the strength of associations. Overall,* MDM2* SNP285 polymorphism was significantly associated with a decreased overall cancer risk with the heterozygous model (OR = 0.89, 95% CI = 0.79–0.99), and reduced ORs were observed with other genetic models (dominant: OR = 0.90, 95% CI = 0.79–1.01 and allele comparison: OR = 0.91, 95% CI = 0.80–1.03) but not reaching statistical significance. Stratification analysis indicated a decreased risk for ovarian cancer, Caucasians, and studies with relatively large sample size. Despite some limitations, this meta-analysis indicated that the* MDM2* SNP285 polymorphism was associated with a decreased cancer risk, which warrants further validation in large and well-designed studies.

## 1. Introduction

Cancer is a major public health problem and about 14.1 million new cancer cases and 8.2 million deaths occurred in 2012 worldwide according to the GLOBOCAN estimates [1]. The development and progression of cancer is a multistage process and alterations of oncogenes, tumor suppressor genes, and stability genes are responsible for tumorigenesis [2]. Tumor suppressor gene* p53* is one of the most frequently mutated genes and numerous studies have reported that the* p53* mutations play an important role in human cancers [3–6].

The* mouse double minute 2* (*MDM2*) gene encodes a protein which could negatively regulate the activity of p53 tumor suppressor protein by binding to the latter and leading to its ubiquitination [7, 8]. Elevated MDM2 levels have been detected in several human cancers even though with wild-type* p53* due to the abnormal expression of* MDM2* gene and/or protein [9, 10], which suggested as an alternative way for p53 inactivation in tumorigenesis [11]. Therefore, polymorphisms and mutations affecting MDM2 expression may contribute to the susceptibility to various cancers.

Two functional SNPs have been discovered which are located in the* MDM2* intronic promoter (P2): SNP309 (rs2279744 T>G) [12] and SNP285 (rs117039649 G>C) [13] located 24 base pairs upstream of SNP309. Compared with the SNP309T allele, the G-variant of SNP309 increases MDM2 transcription through enhancing the binding of the transcription factor Sp1 [12]. In contrast, the presence of SNP285C allele would reduce the binding strength between Sp1 and the* MDM2* promoter in comparison with the SNP285G allele [13]. Although the presence of the SNP285C variants seems to antagonize the effect of SNP309G [13], there are some contradictory studies about* MDM2* SNP285 in different types of cancers [13–22]. One of the possible reasons may be the relatively small sample size in individual published studies. Hence, we performed a meta-analysis to provide a more precise estimation of the relationship between* MDM2* SNP285 polymorphism and cancer risk.

## 2. Materials and Methods

### 2.1. Search Strategy

We systematically searched all relevant articles from PubMed, Embase, and Chinese Biomedical (CBM) databases using the search terms: “*MDM2* or* mouse double minute 2*”, “variant or polymorphism or variation”, and “cancer or carcinoma or tumor or neoplasm” (last search was updated on May 19, 2016). We also checked all references of relevant reviews and eligible articles for additional studies. Only the latest or the largest study would be included in the current meta-analysis if studies were carried out with the same or overlapped subjects.

### 2.2. Inclusion and Exclusion Criteria

Studies included in the meta-analysis had to meet the following criteria: (a) evaluating the association between* MDM2* SNP285 polymorphism and cancer risk; (b) case-control design; (c) written in English or Chinese; (d) providing sufficient data to calculate odds ratios (ORs) with corresponding 95% confidence intervals (CIs).

The following exclusion criteria were used: (a) case-only studies or case reports; (b) conference abstracts, reviews, or meta-analysis; (c) duplicate publications; and (d) no available data reported.

### 2.3. Data Extraction

Two authors (Ping Wang and Meilin Wang) independently extracted data from all the publications according to the inclusion and exclusion criteria. Disagreements were resolved through discussion until a consensus was reached. The following information was extracted from each study: first author's surname, year of publication, country of origin, ethnicity, cancer type, control source, total number of cases and controls, genotype methods, and numbers of cases and controls with the GG, GC, and CC genotypes. Ethnic backgrounds were categorized as Caucasians, Africans, or Mixed which contained more than one ethnic group.

### 2.4. Statistical Analysis

Crude ORs and their corresponding 95% CIs were used to evaluate the strength of associations between* MDM2* polymorphism and cancer risk. The pooled ORs were estimated for* MDM2* SNP285 polymorphism under the dominant model (CC+GC* versus* GG), heterozygous model (GC* versus* GG), and allele comparison (C* versus* G). Chi-square-based *Q* test was used to assess the heterogeneity between studies, and *P* < 0.10 was considered as significant heterogeneity exists. The fixed-effects model (the Mantel-Haenszel method) [23] was used when there was no significant heterogeneity; otherwise, the random-effects model (the DerSimonian and Laird method) [24] would be applied. Potential publication bias was assessed by the funnel plot as described previously [25]. The asymmetry of the funnel plot was evaluated using Egger's linear regression test [26]. Subgroup analysis was performed by cancer type (if one cancer type contained only one study, it would be merged into the “other” group), ethnicity, and sample size (<500 and ≥500). Sensitivity analysis was used to evaluate the effect of individual investigations on the overall cancer risk by excluding each investigation individually and recalculating the ORs and the 95% CIs [27]. All the statistical tests were performed with Stata (version 12.0; Stata Corporation, College Station, TX). All *P* values were two-sided, and a *P* < 0.05 was considered as statistically significant.

## 3. Results

### 3.1. Study Characteristics

**Figure 1:**
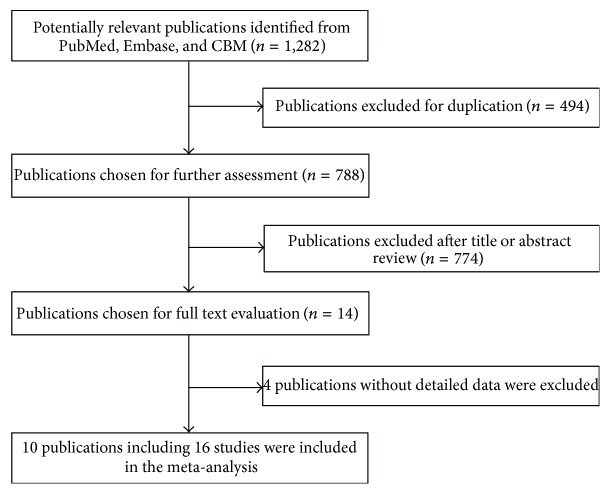
Flowchart of included studies for the association between* MDM2* SNP285 polymorphism and cancer susceptibility.

As shown in Figure 1, a total of 1,282 published records were retrieved by using the search terms described above, consisting of 542 related studies from PubMed, 690 from Embase (494 overlapped studies were deducted), and 50 studies from CBM database. After rigorous assessment of abstracts and contents, only 14 publications met the crude inclusion criteria and were subjected for further evaluation. Of these 14 publications, four were excluded for being without detailed data for further evaluation [28–31], and a total of 10 publications met the inclusion criteria [13–22]. Of the 10 publications, one publication [18] with two ethnic groups was separated as two independent studies, two publications [13, 16] with two cancer types were separated as two independent studies, and one publication [21] with four cancer types was also separated as four independent studies. A total of 10 publications including 16 studies were included in the final meta-analysis (Table 1).

For those studies [13, 15, 16, 21] that used the same control group, the control numbers were only calculated once in the total number of controls, and overlapped controls and cases were subtracted from the total number. Overall, 16 published studies of 14,573 cases and 9,115 controls were included in the final meta-analysis. Of the 16 studies, sample sizes of case ranged from 119 to 2501, in which four studies focused on breast cancer, three on lung cancer, two on ovarian cancer, prostate cancer, and cervical cancer, and others (colon cancer, hepatocellular carcinoma, and endometrial cancer) with only one study. There were 13 studies on Caucasians, two studies on mixed ethnicity, and only one study on Africans. Of all the studies, 14 were population-based, while two were hospital-based, 7 studies with sample size less than 500 and 9 studies with sample size greater than 500. Most of the cases were histologically confirmed and controls were mainly matched for sex, age, and ethnicity.

### 3.2. Meta-Analysis Results

**Figure 2:**
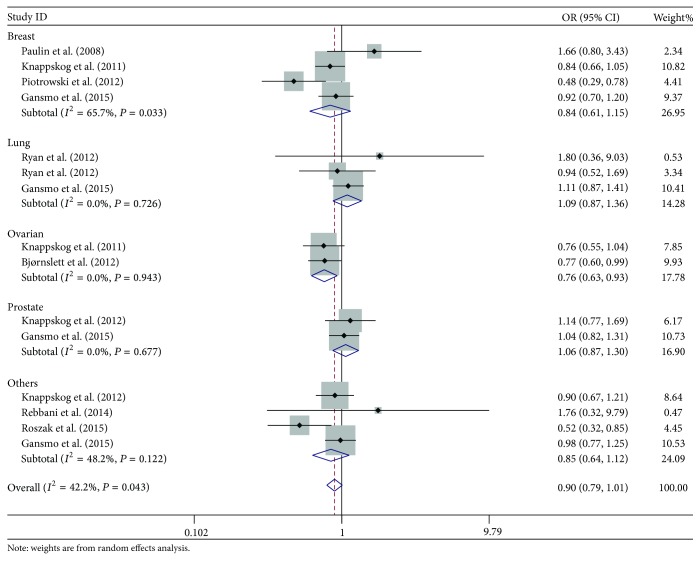
Forest plot of overall cancer risk associated with* MDM2* SNP285 polymorphism by dominant model. For each study, the estimated OR and its 95% CI are plotted with a box and a horizontal line. ⋄, pooled OR and its 95% CIs.

The overall results for the* MDM2* SNP285 polymorphism and cancer risk are shown in Table 2 and Figure 2. We found that there was a significant association between SNP285 polymorphism and overall cancer risk with the heterozygous model (OR = 0.89, 95% CI = 0.79–0.99), and reduced ORs were observed with other genetic models (dominant: OR = 0.90, 95% CI = 0.79–1.01 and allele comparison: OR = 0.91, 95% CI = 0.80–1.03) but not reaching statistical significance. In subgroup analysis by cancer type, a significantly decreased risk was found for ovarian cancer (heterozygous: OR = 0.77, 95% CI = 0.63–0.94; dominant: OR = 0.76, 95% CI = 0.63–0.93; and allele comparison: OR = 0.77, 95% CI = 0.63–0.93), and a much lower OR was observed for breast cancer than that for lung and prostate cancer under all three genetic models. Stratification analysis by ethnicity revealed a statistically significantly decreased cancer risk for Caucasians with the heterozygous (OR = 0.88, 95% CI = 0.79–0.98) and dominant model (OR = 0.89, 95% CI = 0.79–1.00), and reduced OR was found but not reaching statistical significance with the allele comparison (OR = 0.90, 95% CI = 0.79–1.03). In subgroup analysis by sample size, a significantly decreased risk was found for studies with relatively large sample size under the heterozygous model (OR = 0.92, 95% CI = 0.84–1.00), and reduced ORs were observed but not reaching statistical significance under other genetic models (dominant: OR = 0.93, 95% CI = 0.85–1.02 and allele comparison: OR = 0.94, 95% CI = 0.85–1.04).

### 3.3. Heterogeneity and Sensitivity Analysis

Substantial heterogeneities were observed for the* MDM2* SNP285 polymorphism and risk of cancer under the dominant model (*P* = 0.043) and allele comparison (*P* = 0.014), but not under the heterozygous model (*P* = 0.120). Hence, we used the random-effects model to generate wider CIs. Finally, leave-one-out sensitivity analysis indicated that no single study could alter the pooled ORs obviously (data not shown).

### 3.4. Publication Bias

**Figure 3:**
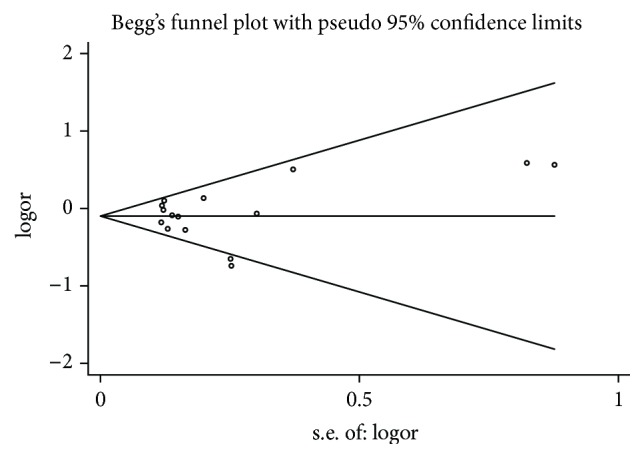
Funnel plot analysis to detect publication bias for SNP285 polymorphism by dominant model. Each point represents a separate study for the indicated association.

The shape of the funnel plot seemed symmetry for the SNP285 polymorphism (Figure 3) and no significant publication bias was detected by Egger's test for SNP285 polymorphism (dominant model: *P* = 0.939; heterozygous model: *P* = 0.997; and allele comparison: *P* = 0.886). These suggested that publication bias might not have severe influence on the results of the current meta-analysis on the association between* MDM2* SNP285 polymorphism and cancer risk.

## 4. Discussion

The association between* MDM2* SNP285 polymorphism and cancer risk has been investigated by several research groups, but the conclusions were inconsistent. The most possible reason for the differences between studies is the small sample size in individual studies, which limits the statistical power to detect the real effects of polymorphism. We performed the current meta-analysis to combine the results of all available studies through a systematic search of relevant literatures, which may be useful for evaluating the contribution of SNP285 polymorphism to cancer. In this meta-analysis, which included 16 published studies of 14,573 cases and 9,115 controls, we found that* MDM2* SNP285 polymorphism was significantly associated with a decreased overall cancer risk in the heterozygous model. Furthermore, the subgroup analysis showed that the association was more evident in the studies of ovarian cancer, Caucasians subjects, and relatively large sample size.

MDM2 encodes a protein that binds to and facilitates the degradation of the p53 tumor suppressor protein via the ubiquitination pathway [32, 33]. The cellular level of MDM2 controls both the p53 and pRb pathways and keeps the balance between growth arrest, cell death, senescence, and apoptosis; disturbances of these processes contribute to malignant transformation of cell [34]. MDM2 protein levels and function are precisely controlled at the transcription, translational, and posttranslation levels. Therefore, SNPs occurring in the* MDM2* gene could potentially dysregulated both transcription and translation.

The* MDM2* SNP285G>C is a newly discovered polymorphism, and this is the first report on the association of this polymorphism and cancer risk. It has been suggested that the* MDM2* SNP285C allele might reduce the risk of female cancers (such as breast, ovarian, and endometrial cancer), due to the estrogen receptor (ESR) reported to be as a transcriptional activator with Sp1 [35] and one of the estrogen receptor binding elements (EREs) in the* MDM2* P2 promoter overlapped with the Sp1 binding site harboring SNP285 [16]. In the present meta-analysis, a significantly decreased risk was found between* MDM2* SNP285 polymorphism and ovarian cancer, and a much lower OR was found for breast cancer than that for male cancers (lung and prostate cancer), which was consistent with previous studies.

There are some limitations in our meta-analysis that remain to be addressed. Firstly, there is a limited number of studies that have actually analysed* MDM2* SNP285 polymorphism with cancer susceptibility and the total sample size was relatively small, which may lead to relatively weak power to detect the real association. Secondly, only published studies were included in this meta-analysis. It may be possible that some related unpublished studies as well as literatures published in languages other than English and Chinese were not included, which might lead to a bias to some extent. Thirdly, in the subgroup analysis, only one study was carried out in Africans. This subgroup did not have enough statistical power for us to investigate the real relationship. Fourthly, genotyping methods were different in each study, which will affect the bias. Additionally, our results were based on unadjusted estimates of ORs without adjustment for individual's age, sex, smoking status, drinking status, environmental factors, and other lifestyles.

In summary, this meta-analysis suggests that* MDM2* SNP285 polymorphism was significantly associated with a decreased overall cancer risk with the heterozygous model. However, large and well-designed studies are warranted to validate our finding. Moreover, further studies estimating the effect of gene-gene and gene-environment interactions may provide a better, comprehensive understanding of the association between* MDM2* SNP285 polymorphism and cancer risk.

## Figures and Tables

**Table 1:** Characteristics of studies included in the meta-analysis.

Surname	Year	Country	Ethnicity	Cancer type	Control source	Genotype method	Number of cases				Number of controls				MAF
							All	GG	GC	CC	All	GG	GC	CC	
Knappskog et al. [13]	2011	Norway and Dutch	Caucasian	Breast	PB	PCR-sequencing	1,973	1,843	130	0	2,518	2,322	196	0	0.039
Knappskog et al. [13]	2011	Norway and Dutch	Caucasian	Ovarian	PB	PCR-sequencing	832	782	50	0	2,518	2,322	196	0	0.039
Paulin et al. [14]	2008	Scotland	Caucasian	Breast	PB	PCR-sequencing	299	278	18	3	275	263	12	0	0.022
Bjørnslett et al. [15]	2012	Norway	Caucasian	Ovarian	PB	PCR-sequencing	1,566	1,471	92	3	2,465	2,274	183	8	0.040
Knappskog et al. [16]	2012	Norway	Caucasian	Endometrial	PB	PCR-sequencing	910	846	63	1	2,465	2,274	183	8	0.040
Knappskog et al. [16]	2012	Norway	Caucasian	Prostate	PB	PCR-sequencing	666	608	55	3	675	623	51	1	0.039
Piotrowski et al. [17]	2012	Poland	Caucasian	Breast	PB	Sequencing	468	444	23	1	550	494	54	2	0.053
Ryan et al. [18]	2012	USA	African	Lung	PB	Taqman	142	139	3	0	253	250	3	0	0.006
Ryan et al. [18]	2012	USA	Caucasian	Lung	PB	Taqman	373	351	21	1	398	373	25	0	0.031
Rebbani et al. [19]	2014	Morocco	Mixed	HCC	PB	PCR-sequencing	119	115	4	0	103	101	2	0	0.010
Vargas-Torres et al. [20]	2014	Brazil	Mixed	Cervical	HB	PCR-RFLP	293	288	5	0	184	184	0	0	NA
Gansmo et al. [21]	2015	Norway	Caucasian	Colon	PB	LightSNiP	1,532	1,430	99	3	3,749	3,495	254	0	0.034
Gansmo et al. [21]	2015	Norway	Caucasian	Lung	PB	LightSNiP	1,331	1,232	98	1	3,749	3,495	254	0	0.034
Gansmo et al. [21]	2015	Norway	Caucasian	Breast	PB	LightSNiP	1,717	1,614	100	3	1,872	1,750	122	0	0.033
Gansmo et al. [21]	2015	Norway	Caucasian	Prostate	PB	LightSNiP	2,501	2,319	175	7	1,877	1,745	132	0	0.035
Roszak et al. [22]	2015	Poland	Caucasian	Cervical	HB	Sanger sequencing	456	430	25	1	481	431	47	3	0.055

HB: hospital-based; PB, population-based; PCR-RFLP: polymerase chain reaction-restriction fragment length polymorphism; HCC: hepatocellular carcinoma; MAF: Minor Allele Frequency; NA: not applicable.

**Table 2:** Meta-analysis of the association between *MDM2* SNP285 polymorphism and cancer risk.

Variables	Number of studies	Heterozygous			Dominant			Allele comparison		
		GC *versus* GG			(CC+GC) *versus* GG			C *versus* G		
		OR (95% CI)	*P* ^het^	*I* ^2^ (%)	OR (95% CI)	*P* ^het^	*I* ^2^ (%)	OR (95% CI)	*P* ^het^	*I* ^2^ (%)
All^1^	15	**0.89 (0.79**–**0.99)**	0.120	31.2	0.90 (0.79–1.01)	0.043	42.2	0.91 (0.80–1.03)	0.014	50.0
Cancer type										
Breast	4	0.81 (0.61–1.08)	0.074	56.8	0.84 (0.61–1.15)	0.033	65.7	0.87 (0.62–1.22)	0.015	71.4
Lung	3	1.07 (0.86–1.34)	0.676	0.0	1.09 (0.87–1.36)	0.726	0.0	1.10 (0.89–1.37)	0.773	0.0
Prostate	2	1.02 (0.84–1.25)	0.664	0.0	1.06 (0.87–1.30)	0.677	0.0	1.10 (0.91–1.34)	0.700	0.0
Ovarian	2	**0.77 (0.63**–**0.94)**	0.903	0.0	**0.76 (0.63**–**0.93)**	0.943	0.0	**0.77 (0.63**–**0.93)**	0.985	0.0
Others^2^	4	0.86 (0.66–1.11)	0.175	39.5	0.85 (0.64–1.12)	0.122	48.2	0.84 (0.63–1.14)	0.084	54.8
Ethnicity										
Caucasian	13	**0.88 (0.79**–**0.98)**	0.088	36.9	**0.89 (0.79**–**1.00)**	0.028	47.7	0.90 (0.79–1.03)	0.008	55.2
African	1	1.80 (0.36–9.03)	—	—	1.80 (0.36–9.03)	—	—	1.79 (0.36–8.93)	—	—
Mixed	1	1.76 (0.32–9.79)	—	—	1.76 (0.32–9.79)	—	—	1.74 (0.32–9.62)	—	—
Sample size										
<500	6	0.79 (0.51–1.21)	0.079	49.4	0.84 (0.52–1.35)	0.030	59.5	0.89 (0.54–1.47)	0.012	65.8
≥500	9	**0.92 (0.84**–**1.00)**	0.525	0.0	0.93 (0.85–1.02)	0.368	8.1	0.94 (0.85–1.04)	0.233	23.6

^1^Vargas-Torres et al. (2014), it was not calculated for these models because the numbers of GC and CC genotypes were zero.

^2^The others included endometrial cancer [16], hepatocellular carcinoma [19], colon cancer [21], and cervical cancer [22].

## References

[1] Torre L. A., Bray F., Siegel R. L., Ferlay J., Lortet-Tieulent J., Jemal A. (2015). Global cancer statistics, 2012. *CA: A Cancer Journal for Clinicians*.

[2] Vogelstein B., Kinzler K. W. (2004). Cancer genes and the pathways they control. *Nature Medicine*.

[3] Ahrendt S. A., Hu Y., Buta M. (2003). p53 mutations and survival in stage I non-small-cell lung cancer: results of a prospective study. *Journal of the National Cancer Institute*.

[4] Miller L. D., Smeds J., George J. (2005). An expression signature for p53 status in human breast cancer predicts mutation status, transcriptional effects, and patient survival. *Proceedings of the National Academy of Sciences of the United States of America*.

[5] Leung W. K., To K.-F., Ng Y.-P. (2001). Association between cyclo-oxygenase-2 overexpression and missense p53 mutations in gastric cancer. *British Journal of Cancer*.

[6] Smith G., Carey F. A., Beattie J. (2002). Mutations in APC, Kirsten-ras, and p53-alternative genetic pathways to colorectal cancer. *Proceedings of the National Academy of Sciences of the United States of America*.

[7] Brooks C. L., Gu W. (2006). p53 ubiquitination: Mdm2 and beyond. *Molecular Cell*.

[8] Xiong S. (2013). Mouse models of Mdm2 and Mdm4 and their clinical implications. *Chinese Journal of Cancer*.

[9] Oliner J. D., Kinzler K. W., Meitzer P. S., George D. L., Vogelstein B. (1992). Amplification of a gene encoding a p53-associated protein in human sarcomas. *Nature*.

[10] Momand J., Jung D., Wilczynski S., Niland J. (1998). The MDM2 gene amplification database. *Nucleic Acids Research*.

[11] Bartel F., Meye A., Würl P. (2001). Amplification of the MDM2 gene, but not expression of splice variants of MDM2 MRNA, is associated with prognosis in soft tissue sarcoma. *International Journal of Cancer*.

[12] Bond G. L., Hu W., Bond E. E. (2004). A single nucleotide polymorphism in the *MDM2* promoter attenuates the p53 tumor suppressor pathway and accelerates tumor formation in humans. *Cell*.

[13] Knappskog S., Bjørnslett M., Myklebust L. M. (2011). The MDM2 promoter SNP285C/309G haplotype diminishes Sp1 transcription factor binding and reduces risk for breast and ovarian cancer in caucasians. *Cancer Cell*.

[14] Paulin F. E. M., O'Neill M., McGregor G. (2008). MDM2 SNP309 is associated with high grade node positive breast tumours and is in linkage disequilibrium with a novel MDM2 intron 1 polymorphism. *BMC Cancer*.

[15] Bjørnslett M., Knappskog S., Lønning P. E., Dørum A. (2012). Effect of the MDM2 promoter polymorphisms SNP309T>G and SNP285G>C on the risk of ovarian cancer in BRCA1 mutation carriers. *BMC Cancer*.

[16] Knappskog S., Trovik J., Marcickiewicz J. (2012). SNP285C modulates oestrogen receptor/Sp1 binding to the *MDM2* promoter and reduces the risk of endometrial but not prostatic cancer. *European Journal of Cancer*.

[17] Piotrowski P., Lianeri M., Rubis B. (2012). Murine double minute clone 2, 309T/G and 285G/C promoter single nucleotide polymorphism as a risk factor for breast cancer: a polish experie. *The International Journal of Biological Markers*.

[18] Ryan B. M., Calhoun K. M., Pine S. R. (2012). MDM2 SNP285 does not antagonize the effect of SNP309 in lung cancer. *International Journal of Cancer*.

[19] Rebbani K., Ezzikouri S., Marchio A., Kandil M., Pineau P., Benjelloun S. (2014). MDM2 285G>C and 344T>A gene variants and their association with hepatocellular carcinoma: a Moroccan case-control study. *Infectious Agents and Cancer*.

[20] Vargas-Torres S. L., Portari E. A., Klumb E. M. (2014). Effects of MDM2 promoter polymorphisms on the development of cervical neoplasia in a Southeastern Brazilian population. *Biomarkers*.

[21] Gansmo L. B., Knappskog S., Hveem K., Vatten L., Lønning P. E. (2015). Influence of MDM2 SNP309 and SNP285 status on the risk of cancer in the breast, prostate, lung and colon. *International Journal of Cancer*.

[22] Roszak A., Misztal M., Sowińska A., Jagodziński P. P. (2015). Murine double-minute 2 homolog single nucleotide polymorphisms 285 and 309 in cervical carcinogenesis. *Molecular Diagnosis & Therapy*.

[23] Mantel N., Haenszel W. (1959). Statistical aspects of the analysis of data from retrospective studies. *Journal of National Cancer Institute*.

[24] DerSimonian R., Laird N. (1986). Meta-analysis in clinical trials. *Controlled Clinical Trials*.

[25] Chen S., Zhu J.-H., Wang F. (2015). Association of the Asp312Asn and Lys751Gln polymorphisms in the *XPD* gene with the risk of non-Hodgkin's lymphoma: evidence from a meta-analysis. *Chinese Journal of Cancer*.

[26] Egger M., Smith G. D., Schneider M., Minder C. (1997). Bias in meta-analysis detected by a simple, graphical test. *British Medical Journal*.

[27] Wang Y.-W., Zhang S.-D., Xue W.-J., Zhu M.-L., Zheng L.-Z. (2015). SHMT1 C1420T polymorphism contributes to the risk of non-Hodgkin lymphoma: evidence from 7309 patients. *Chinese Journal of Cancer*.

[28] Knappskog S., Lønning P. E. (2011). Effects of the MDM2 promoter SNP285 and SNP309 on Sp1 transcription factor binding and cancer risk.. *Transcription*.

[29] Knappskog S., Lønning P. E. (2011). MDM2 promoter SNP285 and SNP309; phylogeny and impact on cancer risk. *Oncotarget*.

[30] Taghizadeh S., Hosseinpour Feizi M. A. (2011). The study of polymorphisms of Mdm2 gene promoter (SNP285, SNP309, SNP344, SNP443) in breast cancer in north-west of Iran. *Cell Journal (Yakhteh)*.

[31] Knappskog S., Gansmo L. B., Dibirova K. (2014). Population distribution and ancestry of the cancer protective MDM2 SNP285 (rs117039649). *Oncotarget*.

[32] Bartel F., Taubert H., Harris L. C. (2002). Alternative and aberrant splicing of *MDM2* mRNA in human cancer. *Cancer Cell*.

[33] Cai X., Yang M. (2012). The functional MDM2 T309G genetic variant but not P53 Arg72Pro polymorphism is associated with risk of sarcomas: a meta-analysis. *Journal of Cancer Research and Clinical Oncology*.

[34] Polager S., Ginsberg D. (2009). P53 and E2f: partners in life and death. *Nature Reviews Cancer*.

[35] Saville B., Wormke M., Wang F. (2000). Ligand-, cell-, and estrogen receptor subtype (*α*/*β*)-dependent activation at GC-rich (Sp1) promoter elements. *Journal of Biological Chemistry*.

